# Biodiversity of Prokaryotic Communities Associated with the Ectoderm of *Ectopleura crocea* (Cnidaria, Hydrozoa)

**DOI:** 10.1371/journal.pone.0039926

**Published:** 2012-06-29

**Authors:** Cristina Gioia Di Camillo, Gian Marco Luna, Marzia Bo, Giuseppe Giordano, Cinzia Corinaldesi, Giorgio Bavestrello

**Affiliations:** 1 Department of Life and Environmental Sciences, Polytechnic University of Marche, Ancona, Italy; 2 Institute of Marine Sciences–National Research Council (CNR), Venice, Italy; 3 Department for the Study of the Land and its Resources, University of Genoa, Genoa, Italy; Argonne National Laboratory, United States of America

## Abstract

The surface of many marine organisms is colonized by complex communities of microbes, yet our understanding of the diversity and role of host-associated microbes is still limited. We investigated the association between *Ectopleura crocea* (a colonial hydroid distributed worldwide in temperate waters) and prokaryotic assemblages colonizing the hydranth surface. We used, for the first time on a marine hydroid, a combination of electron and epifluorescence microscopy and 16S rDNA tag pyrosequencing to investigate the associated prokaryotic diversity. Dense assemblages of prokaryotes were associated with the hydrant surface. Two microbial morphotypes were observed: one horseshoe-shaped and one fusiform, worm-like. These prokaryotes were observed on the hydrozoan epidermis, but not in the portions covered by the perisarcal exoskeleton, and their abundance was higher in March while decreased in late spring. Molecular analyses showed that assemblages were dominated by Bacteria rather than Archaea. Bacterial assemblages were highly diversified, with up to 113 genera and 570 Operational Taxonomic Units (OTUs), many of which were rare and contributed to <0.4%. The two most abundant OTUs, likely corresponding to the two morphotypes present on the epidermis, were distantly related to Comamonadaceae (genus *Delftia*) and to Flavobacteriaceae (genus *Polaribacter*). Epibiontic bacteria were found on *E. crocea* from different geographic areas but not in other hydroid species in the same areas, suggesting that the host-microbe association is species-specific. This is the first detailed report of bacteria living on the hydrozoan epidermis, and indeed the first study reporting bacteria associated with the epithelium of *E. crocea*. Our results provide a starting point for future studies aiming at clarifying the role of this peculiar hydrozoan-bacterial association.

## Introduction

Anthozoan cnidarians are known to host rich populations of associated bacteria: the mucus layers of the hard coral *Porites astreoides* Lamarck, 1816 and those of the zoanthid *Palythoa* sp., for instance, host rich procariotic assemblages, whose abundance is regulated by the self-cleaning mechanisms of the cnidarian host [1], [2]. Bacteria isolated from the ectodermal surface typically produce bioactive compounds, this is the case of the gorgonians *Subergorgia suberosa* (Pallas, 1766) and *Junceella juncea* (Pallas, 1766) [3] and of the soft coral *Dendronephthya* sp. that, by inhibiting the larval settlement, help the host in maintaining its surface clean [4]. Recently, bacteria were also found associated with black corals (such as the wire-coral *Stichopathes lutkeni* Brook, 1889 [5]). While the symbioses between microorganisms and anthozoans have been widely investigated, the interactions between bacteria and hydrozoans are still largely unknown, and this is especially evident for the bacterial assemblages colonizing the epithelia of their hosts.

Hydroids present an exoskeleton made up of polysaccharides and proteins (the perisarc), that can either envelop the zooids or can be limited to stolons and branches. This structure is frequently colonized by a complex assemblage of protists and prokaryotes [6]–[9]. Recently, the presence of chitinolytic bacteria belonging to the genus *Vibrio* has been reported in association with the perisarc of different species of hydroids [10], [11]. Several epibiotic microorganisms were reported using electron microscopy and cultivation approaches on different hydroid species and within the same species in various parts of the colony (stem, branches, hydrothecae, tentacles etc) [12].

Very few cases of bacteria living on the hydrozoan epidermis were until now reported: gram-negative, rod-shaped bacteria were found on the epidermis of the green hydra along the margin of the ectodermal cells and becoming particularly abundant on the hypostome and the foot [13]. TEM analyses also revealed the presence of elongated bacterial cells on the epithelium of *Pennaria disticha* (Goldfuss, 1820) [14].

Several hydrozoan species are known to host bacteria inside their tissues, for example, spirochaetes in *Hydra circumcincta* Schulze, 1914 were localized i) extracellularly, – in the spaces between epitheliomuscular cells, ii) intracellularly – inside vacuoles of the epitheliomuscular cells and iii) through the mesoglea [15]. The gastroderm of some strains of *Hydra viridissima* (Pallas, 1766) contained bacterial vesicles associated with cells containing the symbiotic green alga *Chlorella* sp., that could promote metabolic exchanges between the host and the algal symbionts [16]. The recent discovery of nine species of bacteria living inside the epidermal cells of the hydrozoan *Tubularia indivisa* Linnaeus, 1758 has led to the hypothesis of their involvement in the production of cnidarians toxins [17].

The main caveats of the available studies on the associations between marine invertebrates (including hydrozoa) and bacteria, is that almost all of them have been based only on morphological descriptions based on microscopy or on classical cultivation-based techniques [18] that often led to erroneous identifications. The use of culture independent and highly sensitive techniques (e.g., tag-encoded amplicon pyrosequencing of hypervariable regions of the 16S rRNA gene) allows to overcome these problems and to identify also “rare” prokaryotic taxa, which could play a significant role in these associations [19].

Here we investigated the association between the Anthomedusan hydroid *Ectopleura crocea* (L. Agassiz, 1862) and the prokaryotic assemblages colonizing the hydranth surface and tested the hypothesis that the interaction and characteristics of the microbial assemblages change over time and according to the anatomical regions of the hydranths. We used a combination of electron and epifluorescence microscopy and molecular techniques (the tag-encoded pyrosequencing of the 16S rRNA gene). The application of the high-resolution pyrosequencing technique makes this study the most detailed investigation carried out so far to describe the biodiversity of prokaryotes associated with a marine hydroid.

## Materials and Methods

### Samplings

**Figure 1:**
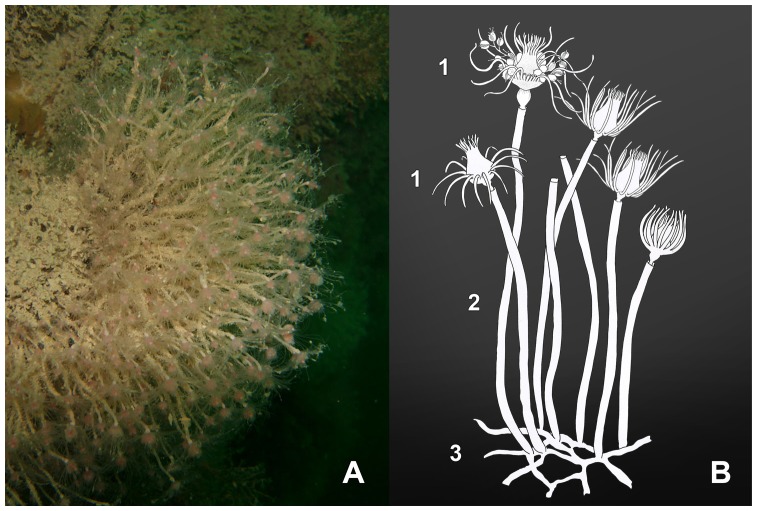
Main characteristics of *Ectopleura crocea*. A) Underwater photograph of the hydroid. B) Scheme of the colony. 1. Feeding and reproductive polyps; 2. Stems (hydrocauli); 3. Tangled stolons anchoring the colony to the substrate (hydrorhizae).

Samplings of *Ectopleura crocea* (Figure 1A, B) were carried out in 2009 and 2010 by SCUBA diving on the starboard bow of the wreck *Nicole* sunk at water depth of 10–12 m about 2 miles off the Conero Promontory (Numana, Italy) (43°30′076′′N – 13°40′191′′E). At each sampling time (April 2009, March 2010, April 2010 and May 2010), three hydroid colonies of about 100 hydranths each were detached from the wreck walls and, using previously sterilized steel forceps, immediately put into sterilized test-tubes in order to avoid microbial contamination. Once back in the laboratory, the samples for microbiological analyses were washed twice in autoclaved and 0.2-µm prefiltered seawater to remove seawater microbes which can influence the composition of the hydrant-associated bacterial assemblage. Hence, the samples were processed according to the protocols required for each specific analysis detailed here below.

### Morphological description and distribution

**Figure 2:**
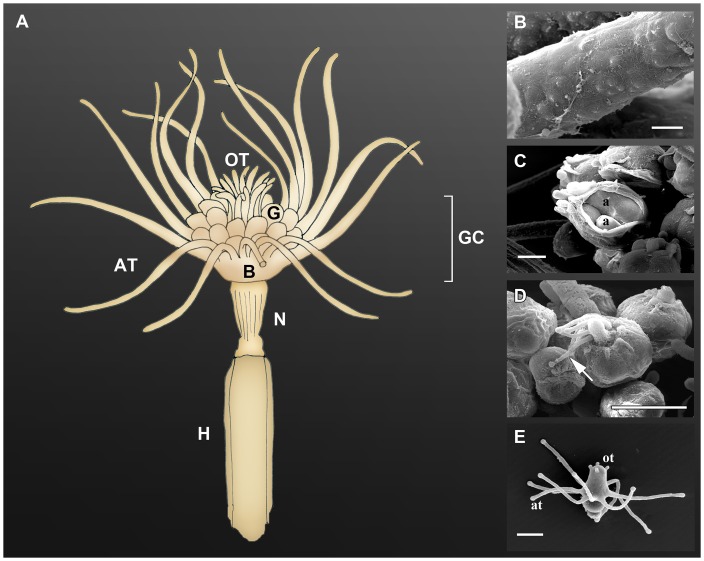
Portions of *E. crocea* examined to find prokaryotes. A) Scheme illustrating the main features of the polyp stage. GC gastric column, AT aboral tentacles, OT oral tentacles, G gonophores, B basal portion of hydranth, N neck zone, H hydrocaulus (portion covered with perisarc). B–E. Close-up view at scanning electron microscope. B). Aboral tentacle colonized by bacteria. C. Broken female gonophore containing immature actinulae round in shape (a). Bacteria were rarely found on actinulae at this stage. D. Mature female gonophore with actinula's tentacles protruding through the opening (white arrow). Bacteria were often observed on these tiny tentacles. E. Released and free-living actinula with developed aboral (at) and oral (ot) tentacles. Scale bars: B 20 μm; C 500 μm D, E 200 μm.

In order to observe, by means of electron microscopy (SEM and FE-SEM), the epibionts' morphology and their distribution on the host, several hydroid polyps were cut from each collected colony with micro-forceps and then fixed for three hours in 2.5% glutaraldehyde buffered with filtered seawater (pH adjusted to 7.5–7.8, with 0.1N NaOH). Then, for the Scanning Electron Microscopy (SEM), part of the samples was washed with distilled water, dehydrated in a graded ethanol series and dried with the Critical Point Dryer. They were then coated with gold-palladium in a Balzer Union evaporator and examined with a Philips XL20 SEM and a FESEM Zeiss Supra 40. Each considered portion of the polyp (oral and aboral tentacles, gastric column, hydranth base, neck, gonophores and hydrocaulus) was analysed by SEM to investigate the bacterial distribution along the colony (Figure 2). To investigate the possible mechanism of transmission of bacteria, we collected and analysed the actinulae just released and the actinulae developed inside the female gonophores. For ultrastructural investigation by Transmission Electron Microscopy (TEM), part of the fixed samples was placed in filtered seawater, then post-fixed in 2% osmium tetroxide for 30 min, dehydrated in a graded ethanol series and embedded in Epon 812. Sections were cut on LKB ultramicrotome and the thin sections (70 nm) were stained with uranyl acetate and lead citrate and observed in a TEM CM200 operating at 100KV.

### Comparison with other geographic areas

During the same study period, three other populations of *E. crocea* collected from different geographic areas were examined under electron microscopy to establish whether the association of microbes was a constant feature in this hydroid species. We analyzed colonies found in the shallow waters of the Murano's channels (Venice, North Adriatic Sea, located approximately 100 nautical miles apart northward from the wreck *Nicole*) and collected from the ship keels. Additional colonies were collected from a floating buoy deployed 10 miles off Brindisi (South Adriatic Sea, located approximately 300 miles apart southward from the wreck *Nicole*). Finally, additional samples were collected from fish farm cages located in the Ligurian Sea (Western Mediterranean Sea, located approximately 400 miles apart from the wreck *Nicole* in a different biogeographic region of the Mediterranean Sea). Other athecate hydroid species, namely *Eudendrium glomeratum* (Picard, 1952) and *Eudendrium racemosum* (Cavolini, 1785), which are common on the wreck *Nicole*, were also examined to verify the presence of associated microbial assemblages using electron microscopy.

### Variations of abundance of the epibiotic prokaryotes over time and along the hydranths using electron microscopy

In the samples collected in March 2010, April 2010 and May 2010, five of the hydranths prepared for SEM were randomly chosen to determine the total microbial abundance (expressed as number of cell mm^−2^). Five pictures of each hydroid portion were taken under the electron microscope; in each photo, bacteria were counted in nine randomly chosen areas having dimension of 10×10 µm^2^. The results were reported according to the examined portion of the hydroid (aboral tentacles, oral tentacles, gonophores, gastric column, base of the hydranth, neck region and hydrocaulus).

### Variations of abundance of the epibiotic prokaryotes over time using epifluorescence microscopy

The abundance of prokaryotes associated with hydrozoan hydranths was determined in April 2009, March 2010 and May 2010 by using epifluorescence microscopy. Ten hydranths from each of the 3 freshly-collected *Ectopleura crocea* colonies were preserved in 1 ml of 0.2 µm pre-filtered formalin (2% concentration in sterile seawater). To check the detachment efficiency of the prokaryotic cells from the hydranths, aliquots from each suspension (i.e., the formalin solution containing the hydrants) were treated with ultrasounds for 0, 1, 3, 8, and 15 min by using a Branson Sonifier 2200 (60W) in an ice bath to prevent overheating. Each replicate was then homogenized, vigorously vortexed and treated with ultrasounds for three times for one minute each, with 30 seconds intervals within each cycle, as this procedure optimized the detachment of prokaryotes from the hydrants.

Aliquots from each of the obtained suspensions were filtered onto 0.2 µm pore-size aluminium oxide filters (Anodisc, Whatman). Filters were stained using SYBR Green I (10000× in anhydrous dimethyl sulfoxide, Molecular Probes) by adding 20 µl of the stock solution (previously diluted 1∶20 with 0.2 µm filtered Milli-Q water). The filters were incubated 15 minutes in the dark, washed twice with 3 ml of sterilized Milli-Q water, then mounted onto microscope slides and added with 20 µl of antifade solution (50% phosphate buffer and 50% glycerol containing 0.5% ascorbic acid). Filters were analyzed by epifluorescence microscopy (Zeiss Axioskop 2, magnification ×1,000) under blue light excitation. For each filter, at least 20 microscope fields were observed. The data were expressed as number of cells per hydrant.

### Metagenetic analysis of the epibiotic prokaryotes

To identify the epibiotic prokaryotes, we used the tag-encoded amplicon pyrosequencing of hypervariable regions (V5 and V6) of the prokaryotic 16S rRNA gene. The genomic DNA was extracted from ten hydrants from freshly-collected *Ectopleura crocea* samples in April 2009 and March 2010. These two samples were selected on the basis of the results of total abundance, which showed that these samples were characterized by the highest abundance of epibiotic prokaryotes. For DNA extraction, the hydrants were put into a sterile tube and the DNA extracted by using the UltraClean Soil DNA Isolation kit (MoBio Laboratoires) following the manufacturer protocol. The concentrations of extracted DNA were determined by using a NanoDrop^TM^ fluorospectrometer (Thermo Scientific) and SYBR Green I as a stain. Extracted DNA was stored at −80°C until further molecular analyses of prokaryotic diversity. Bacterial and archaeal 16S ribosomal DNA (rDNA) amplicons were generated using the universal primers 789F (5′-TAGATACCCSSGTAGTCC-3′) and 1046R (5′- CGACAGCCATGCANCACCT-3′; [20], [21]). All PCR reactions were performed in a volume of 50 µl in a thermalcycler (Biometra, Germany) using the MasterTaq® kit (Eppendorf AG, Germany), which reduces the effects of PCR-inhibiting contaminants. Thirty PCR-cycles were used, consisting of 94°C for 1 minute, 55°C for 1 minute and 72°C for 2 minute, preceded by 3 minutes of denaturation at 94°C and followed by a final extension of 10 minutes at 72°C. To check for eventual contamination of the PCR reagents, negative controls containing the PCR-reaction mixture but without the DNA template were run during each amplification. Positive controls, containing genomic DNA of *Escherichia coli*, were also used. PCR-products were checked on agarose-TBE gel (1%), containing ethidium bromide for DNA staining and visualization. Twelve different reactions were run for each sample and then combined together to reduce possible PCR biases and to reach the amount necessary for 454 analysis. The amplicons were purified using Amicon Ultra 50 k device (Millipore). The amplicon length and concentration were estimated using the BioAnalyzer microfluidics device (Agilent), and then each amplicon was sequenced via emulsion PCR (which was performed using the recommended kit and protocol from 454 Life Sciences) by using a Genome Sequencer FLX Titanium (Roche). The analysis of the 16S rDNA sequences obtained was performed using the RDP's Pyrosequencing Pipeline (http://pyro.cme.msu.edu/ [22]). Briefly, the sequences were firstly analysed using the “Pipeline Initial Process”, in order to trim off the key tag and primers, and to remove sequences of low quality (using a minimum average exp. quality score of 20). Then, bacterial and archaeal sequences were downloaded from each original FASTA sequence file using the option “FASTA Sequence Selection”. Sequences were aligned using the “Pyrosequencing Aligner” tool and clustered using the “Complete Linkage Clustering” tool. Rarefaction curves, the number of OTUs (Operational Taxonomic Units) and the non-parametric Chao1 estimator were determined using the “Analysis tools” available in the RDP pipeline. The taxonomical identification of bacterial and archaeal sequences was performed using the RDP classifier [18], using the default bootstrap cutoff of 80%. The partial 16S rDNA sequences obtained in this study have been deposited in the NCBI Short Read Archive under the accession number SRA052825.3.

### Ethics statement

No specific permits were required for the described field studies. The wreck Nicole is not private, the buoy and the fish farm cage are private, but no specific permissions were required for all the mentioned locations or activities and the sampling procedures employed did not cause damage to the privately-owned properties. The field studies did not involve endangered or protected species and the locations were not protected in any way. The D.P.R. n° 1639 of the 2^th^ October 1968, articles 26 – 29 regards only fish samplings or researches inherent to fishing activities (art. 7). The collection of hydroids is excluded by this kind of regulation. No permits are necessary to collect hydroids.

## Results

*Ectopleura crocea* is a colonial hydroid distributed worldwide in temperate waters. In the study area, this hydroid settles on the wreck *Nicole* showing a marked temporal variation [23]. The hydroid species appears in autumn (November or December, when the *in situ* temperature is ca. 15°C), reaches its highest abundance in April and then declines in late spring (at the end of May or early June, when the sea temperature ranges between 18 and 21°C). Colonies are formed by dense tufts of polyps, each one showing an erect stem – the hydrocaulus – surrounded by perisarc and a hydranth lacking of perisarc and bearing mouth and tentacles (Figure 2A–E).

### Morphological description of prokaryotes and their distribution

**Figure 3:**
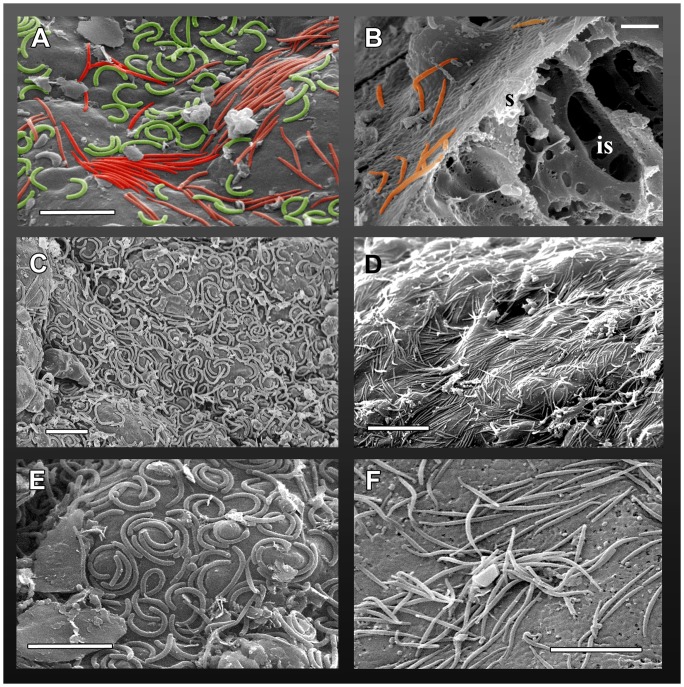
SEM pictures of microorganisms associated with the *Ectopleura crocea* epidermis. A) Surface of a tentacle densely covered with the two morphotypes of microorganisms living on the epidermis: one is horseshoe-shaped (green; named *Type I*) and the other is fusiform, worm-like (red; named *Type II*). B) Portion of a broken tentacle, bacteria were present on the surface (s) but not inside (is). C–D) Enlargements of tentacle portions which were densely covered by the two microorganisms. E) Particular of the horseshoe-shaped *Type I*, showing the peculiar arrangement in the tentacle grooves. F) Particular of a cluster of the worm-like *Type II*. Scale bars: A, C, E, F 5 μm; B 2 μm; D 10 μm.

The electron microscopy clearly revealed the presence of dense populations of prokaryotes living in association with *E. crocea* hydranths. Two microbial morphotypes were observed, both displaying an elongated shape. The first, here named *Type I*, is horseshoe-shaped, 2 to 3.6 µm long (3 µm ±0.17) and 0.2 to 0.3 µm wide (0.2 µm ±0.01) (Figure 3 A, C, E), while the second (named *Type II*) is fusiform, worm-like and measures 2.8 to 5.8 µm in length (4 µm ±0.34) and 0.2 to 0.3 µm in width (0.2 µm ±0.01) (Figure 3 A, B, D, F). SEM micrographs of tentacles' transversal sections showed that these prokaryotes were present only on the external surface, and not inside the epitheliomuscular cells (Figure 3B). Prokaryotes were present all around the epidermis but, particularly, on the aboral tentacles. The two morphotypes were often simultaneously present on the hydroid's surface, but the fusiform, worm-like *Type II* was most commonly observed. Both morphotypes were generally observed on the hydranths and mature gonophores, while they were rarely seen on the neck region and never seen along the hydrocaulus.

*E. crocea* shows a free-moving stage bearing tentacles called actinula (Figure 2E). The SEM analysis of just released actinulae revealed the presence of prokaryotes on their epidermis, but microbial cells were rare and scattered. The early developed actinulae, ovoid and lacking tentacles, found inside the female gonophores (Figure 2C), rarely hosted microbial cells.

**Figure 4:**
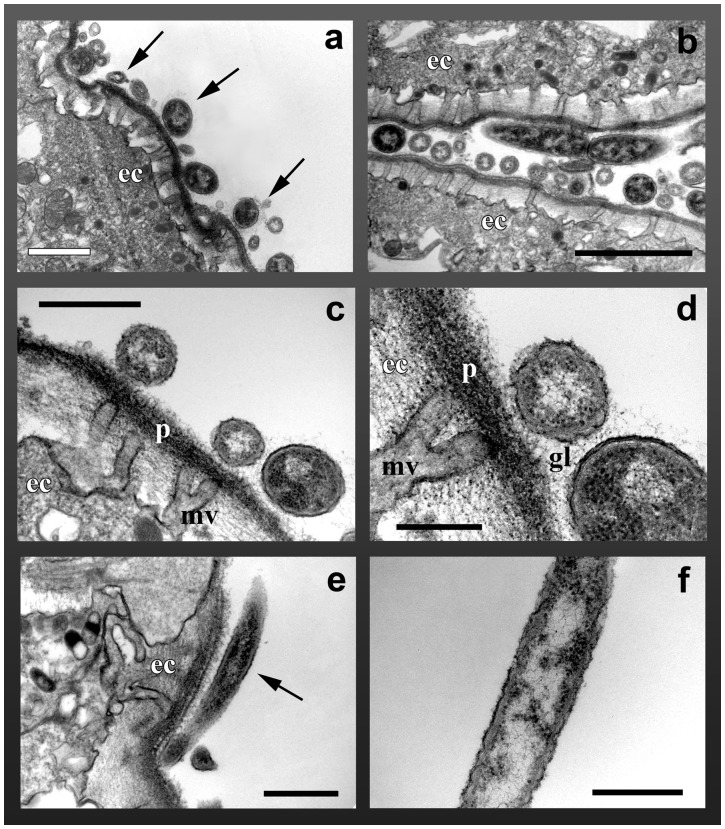
TEM pictures of *Type II* bacteria associated with *Ectopleura crocea.* A) Numerous bacteria in transversal sections (arrows) observed on the hydroid ectoderm (ec). B) Bacteria (longitudinal and tranversal sections) present in a groove of the hydroid ectoderm. C) Bacteria lying on the hydroid periderm (p) are often found in correspondence to the microvilles (mv) of the ectodermal cells. D) Close-up view showing the glycocalyx (gl) surrounded the microorganisms. e–f. Longitudinal section of a bacterium. Scale bars: a, c, e 1 μm; b 2 μm; d, f 0.5 μm.

The transmission electron microscopy (TEM) confirmed that prokaryotes were only on the external surface of the hydranths, directly lying on the hydroid mucoproteinic coating (periderm) (Figure 4A–F). The glycocalyx of the microorganisms kept contact with the hydroid periderm (Figure 4A–F). The hydranth surface observed by means of TEM and SEM did not show any signs of damage due to the presence of microorganisms.

### Variations of abundance of the epibiotic prokaryotes over time and along the hydranths using electron microscopy

**Figure 5:**
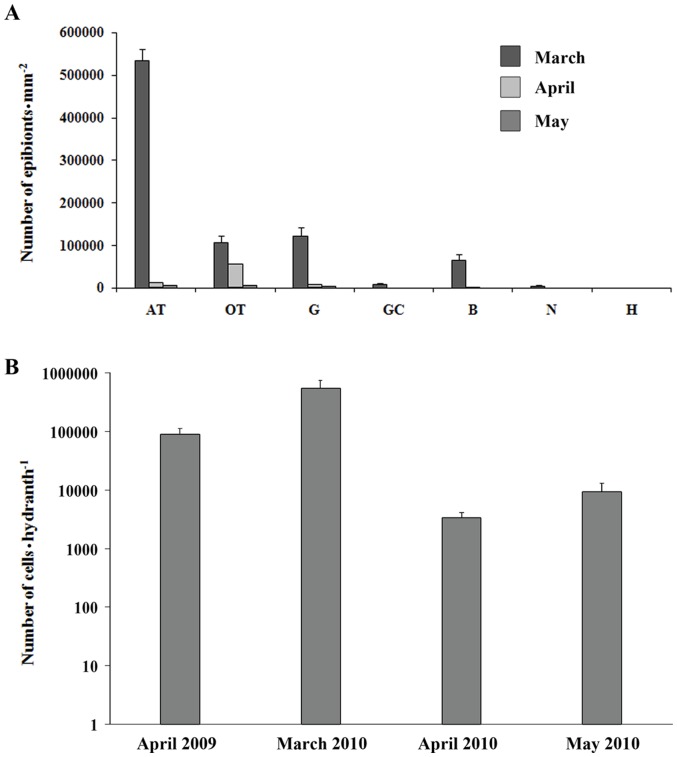
Total prokaryotic abundance, as determined by: A) analysis of the TEM pictures of prokaryotes present on several hydroid portions and B) epifluorescence microscopy. AT: aboral tentacles; OT: oral tentacles; G: gonophores; GC: gastric column; B: base of hydranth; N: neck region; H: hydrocaulus. Standard errors are shown.

The analysis of SEM pictures showed that prokaryotic abundance varied considerably in the different anatomic regions of the hydranth and along the investigated period. The fusiform bacteria (Type II) were the most abundant. The highest density was observed on the aboral tentacles, with more than 500,000 cells mm^−2^, while the lowest values were recorded on the neck region and the hydranth base. Concerning the temporal trend the highest values were observed in March 2010 (when the water temperature was 9.3°C) and the lowest in May 2010 (temperature  = 16.6°C) (Figure 5A). Cell abundance on the aboral tentacles in the period March-May was inversely correlated with temperature (r  = 0,885; p<0.05).

### Variations of abundance of epibiotic prokaryotes over time using epifluorescence microscopy

The abundance of epibionts on *E. crocea*, determined using the epifluorescence microscopy technique, was in the range 3,334±453 to 548,558±113,880 cells per hydrant, with a peak observed in March 2010 (Figure 5B).

### Comparison with Ectopleura from other geographic areas

SEM analyses *of Ectopleura* specimens collected from the Murano's channel and the fish farm cages from the Ligurian Sea displayed the presence of prokaryotes of both types (*Type I* and *II*), despite with a lower density compared with those collected from the wreck *Nicole*. Conversely, the specimens from the floating buoy from Brindisi did not show any associated prokaryote, as well as the other analyzed hydroid species (*E. glomeratum* and *E. racemosum*) from the wreck *Nicole*.

### Metagenetic analysis of the epibiotic prokaryotes

**Figure 6:**
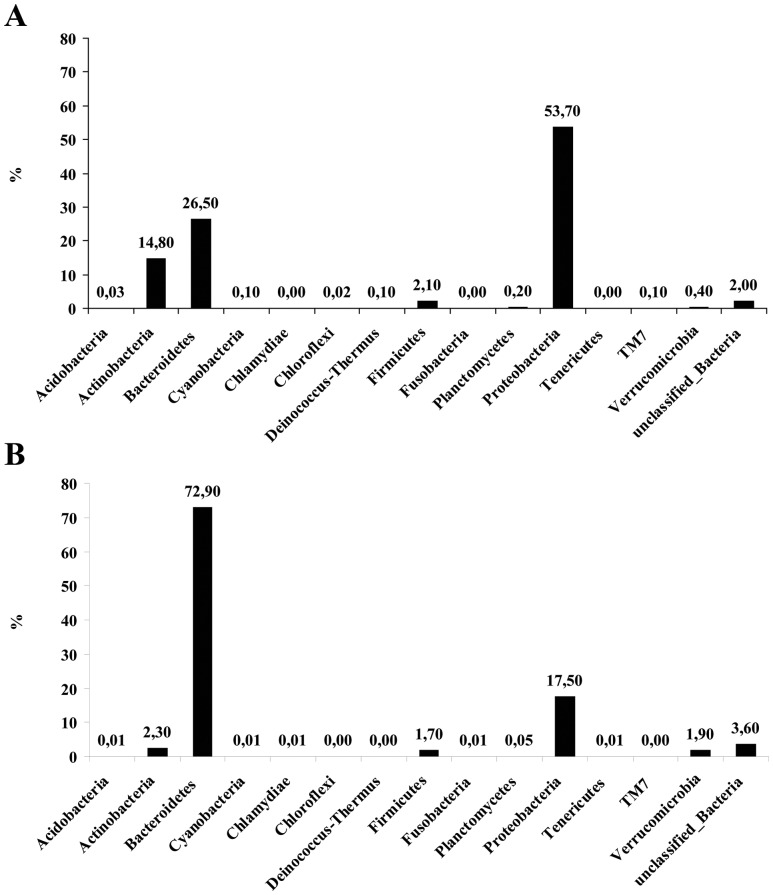
Community composition of bacterial assemblages associated with *E. crocea* in April 2009 (A) and March 2010 (B), as revealed by tag-encoded amplicon pyrosequencing of the 16S rDNA gene. Results are reported at the taxonomic level of phylum.

**Figure 7:**
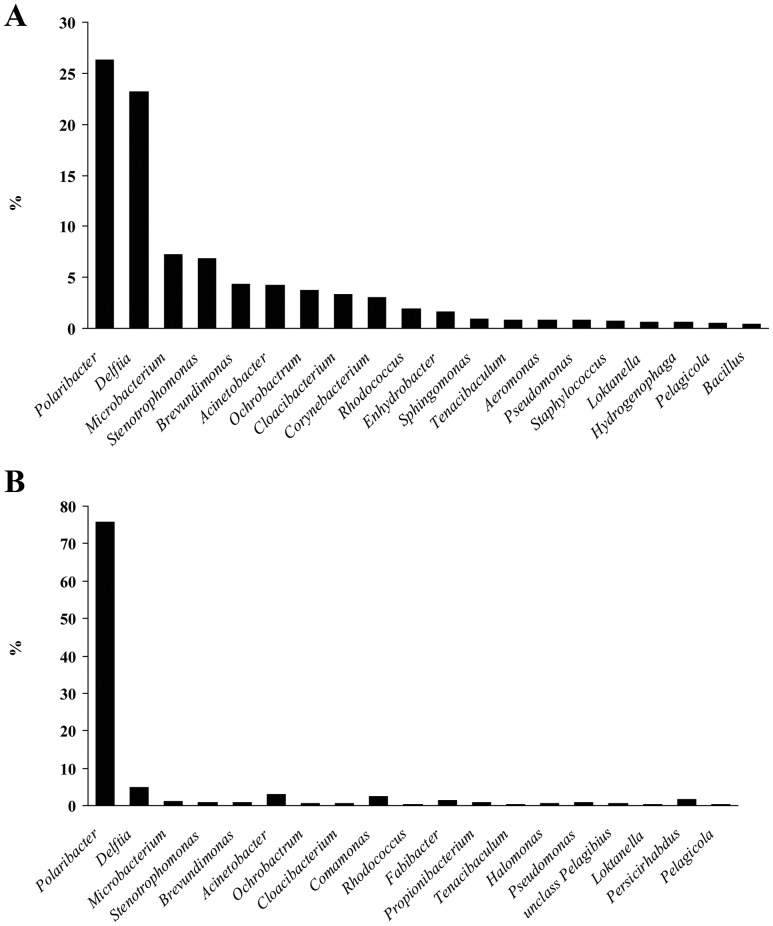
Most abundant bacterial genera associated with *E. crocea* in April 2009 (A) and March 2010 (B), as revealed by tag-encoded amplicon pyrosequencing of the 16S rDNA gene. Reported are the 20 most abundant genera.

The tag-encoded amplicon pyrosequencing analyses of the 16S rRNA gene from the *E. crocea* hydrants resulted in a total of 105,023 reads (32,882 in the samples collected in April 2009 and 72,141 in the samples collected in March 2010), with an average length of 238 and 233 nucleotides (April 2009 and March 2010, respectively) corresponding to a total of 16,324,661 bases sequenced. After filtering out the low-quality and short sequence reads, the number of high quality reads remained was 6,406 and 13,076 (April 2009 and March 2010, respectively). Of these, only 14 and 4 (respectively) matched with Archaea, indicating that the prokaryotic assemblages were largely dominated by Bacteria. In either April 2009 and March 2010, the prokaryotic assemblages were dominated by bacteria belonging to the phyla Proteobacteria and Bacteroidetes (Figure 6A and B), which together accounted for 80–90% of the assemblage (April 2009 and March 2010, respectively). Actinobacteria was the third more represented phylum (15 and 2%, respectively), followed by Firmicutes (both ca 2%). Unclassified bacteria accounted for ca. 2 and 4%, respectively. Rarefaction analyses, based on OTUs definition at 97% similarity, indicated that rarefaction curves almost reached the plateau, and that the diversity was well described with the sequencing effort here utilized (data not shown). Further evaluation of the community structure at finer taxonomical level showed, using the 97% similarity cut-off, the presence of 112 bacterial genera (Figure 7A and B) and 570 bacterial OTUs in the sample collected in April 2009, and of 113 genera and 522 bacterial OTUs in the sample collected in March 2010. In both samples, the assemblage was dominated by two clusters of sequences matching with two genera (Figure 7A and B, reporting the 20 most abundant bacterial genera). In April 2009, 23% of sequences clustered within the family Comamonadaceae, genus *Delftia* (average similarity 91%) while 26% of sequences clustered within the family Flavobacteriaceae (100% similarity) and showed the highest match with sequences of the genus *Polaribacter*. However, the similarity was very low (on average 50%), suggesting that it is probably a new genus/species. In March 2010, the *Polaribacter* cluster accounted for 76% of sequences and the *Delftia* cluster accounted for 5% of sequences. The remaining sequences belonged to many bacterial genera, most of which were “rare” and accounted for a minor percentage within the assemblage (from 0.2% – 0.4% down to 0.01 – 0.02%; see Table S1 for a complete list).

## Discussion

The study of prokaryotic assemblages associated with cnidarians, which belong to one of the earliest branches in the animal tree of life, is potentially important to better understand the specificity of their interactions, their evolution and influence on the cnidarian health [24]. This study, based for the first time on the use of massive parallel sequencing, has enabled the accurate identification of the bacterial assemblages associated with their cnidarian host. The hydroid *E. crocea* represents a mosaic of microenvironments that can be colonized by several organisms, both eukaryotic and prokaryotic, that generally settle only on those portions covered by the exoskeleton [6]–[11], [25]. Several hydroid species have been shown to live with an associated microbial flora [26], but this is the first study dealing with the presence of bacteria on the hydroid's epidermis documented with a molecular approach combined with electron microscopy. Our molecular analyses revealed that most of the observed prokaryotic diversity was within the domain of Bacteria, whereas Archaea accounted for a negligible fraction. The bacterial assemblage was highly diversified, and comprised more than 100 bacterial genera and more than 500 taxa (OTUs) per single hydroid. This high bacterial biodiversity is unprecedented for hydrozoans, and is due to the application of the high resolution tag-encoded amplicon pyrosequencing technique, as already reported for other marine organisms [18], [19], [27]. This high diversity includes a large proportion of “rare” bacterial species, which may be transient microbes or may represent specifically associated microbes, which may provide benefits to their hydroid host (e.g. preventing fouling by other organisms, excluding potential pathogens and resource competitors, providing nutrients).

The high bacterial diversity observed through the metagenetic analysis was associated to two morphotypes revelaed by the electron microscopy analyses, which we named *Type I* and *Type II*. The elongated, worm-like morphotype (*Type II*) was most frequently observed and more abundant when compared to the other type. The two morphotypes were found exclusively on the epidermis of the hydranths and actinulae of *E. crocea*. Portions covered by the thick and stiff perisarcal exoskeleton, such as the stem and the hydrorhiza, were never colonized by these bacteria. The morphotypes *Type I* and *II* were rarely found on the neck, the transition region between the stem and the hydranth, covered with a very thin and soft skeletal layer, probably having a different protein/chitin ratio and therefore partially allowing the epibiosis. This non-random association between the host and the microbes suggests that the two microbes may play a role in the hydroid life and survival.

Molecular analyses indicated the dominance of two OTUs, one clustering within the family Flavobacteriaceae, genus *Polaribacter*, and the other within the family Comamonadaceae, genus *Delftia.* The OTU related within the Flavobacteriaceae family dominated the two libraries, especially the one carried out on the samples collected in March 2010. The similarity with the genus *Polaribacter* was very low (on average 50%), suggesting that this OTU possibly represents a new genus within the Flavobacteriaceae family, or a new species within the *Polaribacter* genus, but further investigations are needed to confirm this hypothesis. The family Flavobacteriaceae is widely distributed in the marine environment, and several members of this family display a cellular worm-like morphology [28], similar to the one displayed by the morphotype *Type II*. In addition, some *Polaribacter* include psycro- or meso-philic species, which do not tolerate high temperatures [29], and this could partly explain the negative relationship observed between sea water temperature and *Type II* abundance on the hydroid surface. Nonetheless, the abundance of this bacterium varied during the study period, showing a peak in March and decreasing in late spring. This event anticipates the subsequent *E. crocea* decrease which, in the North Adriatic Sea, typically occurs in June (till October when the resting stages of the quiescent hydrorhizas give rise to new colonies [23]). While it is possible that the hydroid regression can be triggered by environmental factors, such as the progressive temperature increase [23], the decline of *E. crocea* following the disappearance of the *Polaribacter* species lets to hypothesize that the hydroid might remain susceptible of infections when not protected by its own associated flora. However, further studies are needed to clarify this hypothesis.

The OTU belonging to the family Comamonadaceae with the highest similarity with the genus *Delftia* could be associated to the *Type I* morphotype. This bacterium was less frequently observed compared with the *Type II* morphotype, and this was also reflected in the abundance of this OTU in the two pyrosequencing libraries. Members of this genus are typically marine bacteria and are associated to marine invertebrates, such as sponges or crustaceans. Several *Delfia* spp. are reported to have a curved-rod, horseshoe morphologies [30]. Further cultivation analyses aimed at isolating *Delphia* sp. and *Polaribacter* (cfr) sp. will allow the identification at the species level and the investigation of their functional role within the host.

SEM analyses indicated that epibiontic bacteria were present on *E. crocea* samples collected from different geographic areas, but were absent in other hydroid species of the same areas. This suggests the presence of a species-specific relationship, similar to the one described for *Hydra* [24], and supports the hypothesis that these two species are resident species. This is also confirmed by molecular analyses conducted in different periods (March and April), which indicate a potential selection operated by *E. crocea* on the associated bacterial assemblage. In this regard it has been recently shown that *Hydra* is able to distinguish between the epibionts and other potentially pathogenic prokaryotes colonizing its surface [31].

The morphotypes *Type I* and *Type II* were also observed on the newly released actinulae, suggesting the presence of a vertical transmission of the prokaryotes. While studying the reproductive structures of *E. crocea*, it is common to observe some of the actinula tentacles protruding from the gonophore before the release (Figure 2D). This lets to hypothesize that the bacterial colonization of the *E. crocea* hydrants can occur when prokaryotes, present on the gonophore surface, colonize the protruding tentacles of the actinulae or because of accidental contact between actinula tentacles and other portions of the hydranth. However, additional analyses are needed to test for the ability of the actinulae to collect these bacteria from the surrounding seawater.

It is likely that other similar associations between bacteria and hydroids occur, especially within the family Tubulariidae. However, the investigation of prokaryotes-invertebrate associations in the marine environments using modern molecular techniques, is still in its infancy, with most of the studies being referred to Leptomedusan species [6]–[11], [32]. Since the three genera of hydrozoans showing association with bacteria – *Ectopleura, Hydra* and *Pennaria* – are all included in the order Capitata [33], we hypothesize that this group of marine invertebrates has the ability to establish specific microbe-host interactions.

Bacteria can interact with cnidarian epidermis in several ways. There is a mutualistic relationship between prokaryotes and *Hydra* polyps: while the prokaryotes obtain nutritional benefits from their host, the hydroid partially loses the ability to intake phosphate (used by symbiotic chlorellae) when bacteria are removed [13]. Non-symbiotic *Hydra* species were not able to bud under bacteria-free conditions, suggesting that associated prokaryotes could provide a budding factor [34]. Many coral species are able to produce antifouling molecules, and several bacteria species are known to be directly involved in the biosynthesis of these secondary metabolites [35]. Several coral-associated bacteria grow in the coral mucus layer [36] and produce antimicrobial metabolites, that are believed to protect the coral host from pathogens [2]. It is also possible that the bacteria colonizing the surface of *Ectopleura* release substances that discourage predation. The nudibranch *Cuthona gymnota* (Couthouy, 1838), for instance, feeds on the hydroid piercing the stems while never eats the hydranths. However, hydroid cnidocysts can represent an important deterrent toward predators [37]. This study is one of the first reports of bacteria living on hydrozoan epidermis, and indeed the first reporting a stable association of bacteria with the epithelium of *E. crocea*. Our results represent a starting point for future studies, which may provide insights on the significance and the role of this specific association between bacteria and hydrozoa.

## Supporting Information

Table S1 **List of “rare” bacterial genera associated with** ***E. crocea*** **in April 2009 and March 2010, as revealed by tag-encoded amplicon pyrosequencing of the 16S rDNA gene.** Reported are those genera which are not shown in Figure 7.
(DOC)
