# Supplementary material for: Biodiversity of Prokaryotic Communities Associated with the Ectoderm of Ectopleura crocea (Cnidaria, Hydrozoa)
Source: PLoS One. 2012 Jun 29;7(6):e39926. doi: 10.1371/journal.pone.0039926 (PMC3386928; doi:10.1371/journal.pone.0039926)
Supplement: Table S1 — List of “rare” bacterial genera associated with E. crocea in April 2009 and March 2010, as revealed by tag-encoded amplicon pyrosequencing of the 16S rDNA gene. Reported are those genera which are not shown in Figure 7. (DOC) [file pone.0039926.s001.doc]

**Supplementary Table S1.** List of “rare” bacterial genera associated with *E. crocea* in April 2009 and March 2010, as revealed by tag-encoded amplicon pyrosequencing of the 16S rDNA gene. Reported are those genera which are not shown in Figure 7.

April 2009

| **Genus** | **Relative abundance (%)** |
| --- | --- |
| *Streptococcus* | 0.38 |
| *Polaribacter* | 0.34 |
| *Hellea* | 0.28 |
| *Hydrogenophilus* | 0.26 |
| *Arthrobacter* | 0.22 |
| *Brevibacterium* | 0.20 |
| *Pelomonas* | 0.18 |
| *Phyllobacterium* | 0.16 |
| *Geobacillus* | 0.16 |
| *Methylobacterium* | 0.14 |
| *Fluviicola* | 0.14 |
| *Sphingobium* | 0.12 |
| *Arcobacter* | 0.12 |
| *Lutibacter* | 0.12 |
| *Actibacter* | 0.12 |
| *Aestuariicola* | 0.10 |
| *Rubritalea* | 0.10 |
| *Winogradskyella* | 0.08 |
| *Actinomyces* | 0.08 |
| *Anaerococcus* | 0.06 |
| *Desulfotalea* | 0.06 |
| *Spongiibacter* | 0.06 |
| *Bordetella* | 0.06 |
| *Eudoraea* | 0.06 |
| *Lacinutrix* | 0.04 |
| *Nevskia* | 0.04 |
| *Paracoccus* | 0.04 |
| *Phenylobacterium* | 0.04 |
| *Tateyamaria* | 0.04 |
| *Zimmermannella* | 0.04 |
| *Rhizobium* | 0.04 |
| *Rothia* | 0.02 |
| *Vibrio* | 0.02 |
| *Wautersiella* | 0.02 |
| *Meiothermus* | 0.02 |
| *Methylotenera* | 0.02 |
| *Nitrospira* | 0.02 |
| *Ralstonia* | 0.02 |
| *Aerococcus* | 0.02 |
| *Granulicatella* | 0.02 |
| *Halomonas* | 0.02 |
| *Herbaspirillum* | 0.02 |
| *Lutimonas* | 0.02 |
| *Marinomonas* | 0.02 |
| *Pseudoalteromonas* | 0.02 |
| *Serratia* | 0.02 |
| *Sphingobacterium* | 0.38 |
| *Antarctobacter* | 0.34 |
| *Actinobacillus* | 0.28 |
| *Atopococcus* | 0.26 |
| *Chryseobacterium* | 0.22 |
| *Dechloromonas* | 0.20 |
| *Enterococcus* | 0.18 |
| *Escherichia/Shigella* | 0.16 |
| *Flavobacterium* | 0.16 |
| *Gaetbulibacter* | 0.14 |
| *GpIIa* | 0.14 |
| *Mucilaginibacter* | 0.12 |
| *Phaeobacter* | 0.12 |
| *Sulfitobacter* | 0.12 |
| *Thalassobacter* | 0.12 |
| *Buttiauxella* | 0.10 |
| *Alcaligenes* | 0.10 |
| *Alloiococcus* | 0.08 |
| *Aquabacterium* | 0.08 |
| *Balneimonas* | 0.06 |
| *Blastopirellula* | 0.06 |
| *Brumimicrobium* | 0.06 |
| *Cupriavidus* | 0.06 |
| *Desulfobulbus* | 0.06 |
| *Desulfosarcina* | 0.04 |
| *Ilumatobacter* | 0.04 |
| *Kocuria* | 0.04 |
| *Lactobacillus* | 0.04 |
| *Maribacter* | 0.04 |
| *Maribius* | 0.04 |
| *Methyloversatilis* | 0.04 |
| *Morganella* | 0.02 |
| *Naxibacter* | 0.02 |
| *Paenibacillus* | 0.02 |
| *Pantoea* | 0.02 |
| *Parvibaculum* | 0.02 |
| *Pedomicrobium* | 0.02 |
| *Petrobacter* | 0.02 |
| *Rhodocista* | 0.02 |
| *Ruegeria* | 0.02 |
| *Solirubrobacter* | 0.02 |
| *Sphingosinicella* | 0.02 |
| *Sulfurovum* | 0.02 |
| *Xanthobacter* | 0.02 |
| *Xanthomonas* | 0.02 |

March 2010

| **Genus** | **Relative abundance (%)** |
| --- | --- |
| *Shewanella* | 0.16 |
| *Crocinitomix* | 0.13 |
| *Sphingomonas* | 0.12 |
| *Pseudoalteromonas* | 0.10 |
| *Enhydrobacter* | 0.10 |
| *Streptococcus* | 0.10 |
| *Staphylococcus* | 0.10 |
| *Aeromonas* | 0.10 |
| *Proteus* | 0.10 |
| *Coraliomargarita* | 0.09 |
| *Sphingobium* | 0.09 |
| *Methylobacterium* | 0.09 |
| *Ralstonia* | 0.09 |
| *Hydrogenophaga* | 0.08 |
| *Vagococcus* | 0.07 |
| *Lutimonas* | 0.06 |
| *Arcobacter* | 0.06 |
| *Vibrio* | 0.06 |
| *Colwellia* | 0.06 |
| *Corynebacterium* | 0.05 |
| *Granulicatella* | 0.05 |
| *Bordetella* | 0.05 |
| *Persicirhabdus* | 0.04 |
| *Phyllobacterium* | 0.04 |
| *Anaerococcus* | 0.03 |
| *Gemella* | 0.03 |
| *Aureispira* | 0.03 |
| *Maribacter* | 0.03 |
| *Pelagibacter* | 0.03 |
| *Hellea* | 0.03 |
| *Neisseria* | 0.03 |
| *Massilia* | 0.03 |
| *Acidovorax* | 0.03 |
| *Francisella* | 0.03 |
| *Francisella* | 0.03 |
| *Thalassomonas* | 0.03 |
| *Blastopirellula* | 0.03 |
| *Zimmermannella* | 0.03 |
| *Peptoniphilus* | 0.03 |
| *Enterococcus* | 0.03 |
| *Aestuariicola* | 0.03 |
| *Winogradskyella* | 0.03 |
| *Desulfotalea* | 0.03 |
| *Devosia* | 0.03 |
| *Ruegeria* | 0.03 |
| *Hydrogenophilus* | 0.03 |
| *Pelomonas* | 0.03 |
| *Psychromonas* | 0.03 |
| *Marinomonas* | 0.03 |
| *Cobetia* | 0.03 |
| *Pseudonocardia* | 0.02 |
| *Micrococcus* | 0.02 |
| *Tessaracoccus* | 0.02 |
| *Fusibacter* | 0.02 |
| *Prevotella* | 0.02 |
| *Parabacteroides* | 0.02 |
| *Chryseobacterium* | 0.02 |
| *Zeaxanthinibacter* | 0.02 |
| *Tateyamaria* | 0.02 |
| *Thauera* | 0.02 |
| *Burkholderia* | 0.02 |
| *Duganella* | 0.02 |
| *Serratia* | 0.02 |
| *Fusobacterium* | 0.01 |
| *Gp6* | 0.01 |
| *Rhodopirellula* | 0.01 |
| *Bacillariophyta* | 0.01 |
| *Ilumatobacter* | 0.01 |
| *Mycobacterium* | 0.01 |
| *Arthrobacter* | 0.01 |
| *Acetobacterium* | 0.01 |
| *Veillonella* | 0.01 |
| *Paenibacillus* | 0.01 |
| *Geobacillus* | 0.01 |
| *Mycoplasma* | 0.01 |
| *Sphingobacterium* | 0.01 |
| *Reichenbachiella* | 0.01 |
| *Lutibacter* | 0.01 |
| *Desulfovibrio* | 0.01 |
| *Sulfurimonas* | 0.01 |
| *Pleomorphomonas* | 0.01 |
| *Sulfitobacter* | 0.01 |
| *Phaeobacter* | 0.01 |
| *Thalassobius* | 0.01 |
| *Antarctobacter* | 0.01 |
| *Thalassobacter* | 0.01 |
| *Phenylobacterium* | 0.01 |
| *Methylotenera* | 0.01 |
| *Tepidimonas* | 0.01 |
| *Achromobacter* | 0.01 |
| *Photobacterium* | 0.01 |
| *Amphritea* | 0.01 |
